# Pelvic Congestion Syndrome: The Gynecological Perspective

**DOI:** 10.3390/jcm15041655

**Published:** 2026-02-22

**Authors:** Christian Krambeck, Karolin Tesch, Rafał Watrowski, Nicolai Maass, Ibrahim Alkatout

**Affiliations:** 1 Department of Obstetrics and Gynecology, Kiel School of Gynaecological Endoscopy, University Hospitals Schleswig-Holstein, Campus Kiel, Arnold-Heller Str. 3, House C, 24105 Kiel, Germany; nicolai.maass@uksh.de; 2 Department of Radiology, University Hospitals Schleswig-Holstein, Campus Kiel, Arnold-Heller Str. 3, House C, 24105 Kiel, Germany; karolin.tesch@uksh.de; 3 Department of Gynecology, Helios Hospital Müllheim, Heliosweg 1, 79379 Müllheim, Germany; rafal.watrowski@gmx.at; 4 Faculty of Medicine, University of Freiburg, 79106 Freiburg, Germany

**Keywords:** pelvic congestion syndrome, chronic pelvic pain, pelvic venous insufficiency, transvaginal ultrasound, endometriosis, primary gynecological care

## Abstract

**Background/Objectives:** Chronic pelvic pain (CPP) is defined as pelvic pain lasting longer than six months and is a common yet often overlooked condition, affecting over 40% of women worldwide and accounting for about 10% of gynecological consultations. Despite extensive investigation, including laparoscopy, no cause is identified in up to half of cases. Pelvic congestion syndrome (PCS), also referred to as pelvic venous insufficiency (PVI), has been estimated to account for up to 30% of CPP cases, although it remains underdiagnosed. PCS is caused by venous reflux or obstruction in pelvic veins and is characterized by dull, aching pain worsened by standing, intercourse, post-orgasm, and the premenstrual period. It occurs predominantly in premenopausal women, often after pregnancy. This narrative review aims to improve understanding of PCS and provide practical guidance to support diagnosis and treatment in routine gynecologic practice. **Methods:** We performed a comprehensive review of the current literature focusing on the clinical presentation, pathophysiology and diagnostic and treatment performance of various modalities. Special emphasis was placed on identifying accessible, non-interventional tools suitable for primary gynecological care. **Results:** PCS, CPP and endometriosis exhibit significant clinical overlap, including dysmenorrhea, dyspareunia and chronic pain. However, pathognomonic features like post-coital pain and pain-exacerbation by prolonged standing, combined with specific ultrasound markers, allow for early differentiation. While laparoscopy is often used to investigate CPP, it has limited sensitivity for PCS due to CO_2_-pneumoperitoneum-induced venous compression, and Trendelenburg position, compared to venography, the diagnostic gold standard. In contrast, transvaginal ultrasound (TVUS) serves as a potent first-line tool. Key diagnostic criteria include ovarian vein diameter (>7–8 mm), low flow velocity (<3 cm/s), and myometrial vein dilatation (>5 mm). Furthermore, the frequent co-occurrence of endometriosis and PCS requires a multimodal diagnostic approach to avoid “diagnostic bias.” **Conclusions:** To improve patient outcomes and reduce diagnostic delay, office-based gynecologists should integrate specific vascular TVUS into the routine workup of CPP, not only to diagnose endometriosis but also to identify PCS. Future efforts should focus on standardized TVUS protocols and interdisciplinary care pathways involving gynecologists and interventional radiologists to enable integrated diagnostic and therapeutic approaches for patients with coexisting endometriosis and PCS, addressing both surgical and non-surgical options, as well as the bidirectional relationship and mutual pathophysiological influence between these entities.

## 1. Introduction

Chronic pelvic pain (CPP) is defined as cyclic or non-cyclic pelvic pain for longer than six months, causing functional disability or limitation in activities of daily activities [1]. CPP is a common and frequently overlooked condition in women, with prevalence rates reported as high as 43.4% worldwide [2]. In the United States, the direct annual healthcare costs associated with CPP range from US$193 to US$2457 per woman, with total yearly costs estimated at approximately US$2.8 billion [3]. Approximately 10% of all gynecological consultations maybe related to CPP [2]. Up to forty percent of all laparoscopies are performed due to CPP [4,5], with no explanation for the symptoms being found in 50% of cases [6].

Pelvic congestion syndrome is estimated to be a cause of CPP in up to 30% of cases [7,8,9]. In this manuscript, the term pelvic congestion syndrome (PCS) will be used, as it remains the most commonly applied terminology in gynecological practice, although pelvic venous insufficiency (PVI) or pelvic venous disorders (PeVD) represent more contemporary and pathophysiologically descriptive terms [10]. It is characterized by CPP resulting from venous reflux or obstruction in the pelvic veins [11]. Common symptoms include dull, aching pain exacerbated by standing, intercourse (dyspareunia), post-orgasm, and premenstrually [12]. PCS tends to occur more frequently after pregnancy and is observed almost exclusively in premenopausal women [13]. Although PCS accounts for a substantial proportion of CPP cases, it remains relatively under-diagnosed in gynecologic practice [14]. A central dilemma in the management of PCS is that patients are frequently seen first by gynecologists, who lack the knowledge [14], diagnostic and therapeutic tools needed for the reliable identification of PCS. As a result, a substantial number of women undergo unnecessary diagnostic laparoscopies, which fail to yield meaningful findings in 40–60% of cases [15,16]. Conversely, interventional radiologists possess the expertise required to diagnose and treat PCS, yet are often unfamiliar with the clinical presentation and differential diagnosis of CPP. This discrepancy creates a care gap that delays diagnosis and leads to ineffective treatment.

The limited number of publications on PCS in gynecological journals also suggests a knowledge gap regarding PCS in this field [14]. Another major reason is the difficulty to distinguish it from numerous other conditions. This review explores the challenges in diagnosing CPP in gynecological practice, with a particular focus on the under-recognition and suboptimal diagnosis of PCS and aims to provide practical guidance to support improved diagnostic evaluation and therapeutic decision-making in routine gynecologic care.

## 2. Materials and Methods

### 2.1. Search Strategy and Data Sources

This article is a narrative review designed to synthesize the gynecological perspective on Pelvic Congestion Syndrome (PCS). A comprehensive and iterative literature search was conducted across the PubMed and Google Scholar databases. The search strategy utilized a combination of specific keywords, including: “Pelvic Venous Insufficiency”, “Pelvic Congestion Syndrome”, “Pelvic Venous Disorders”, and “Pelvic Congestion Syndrome Endometriosis”. No date restrictions were applied.

### 2.2. Inclusion Criteria and Selection Process

To ensure clinical relevance and a high level of evidence, the selection process followed a multi-step approach:

Primary Search: Identification of peer-reviewed original research, systematic reviews, and international clinical guidelines (e.g., ESHRE, DGGG, UIP) published in English and German.

Targeted Backward Snowballing: A core element of the methodology involved manual screening of the reference lists from reviews and established clinical guidelines, specifically the German AWMF S2k Guideline on CPP and the ESHRE Guideline on Endometriosis. This “backward snowballing” strategy was employed to identify foundational primary studies and original research regarding differential diagnoses and co-existing pathologies that were not captured by initial database queries.

Specialized Review: The general search was conducted by the first author (C.K.), while the section on Magnetic Resonance Imaging (MRI) was independently synthesized by a radiological expert (K.T.) to ensure technical accuracy.

Inclusion criteria focused on: (1) diagnostic criteria (TVUS and MRI), (2) clinical appearance of PCS, and (3) the gynecological management of CPP and its comorbidities. Case reports were included only if they provided unique insights into rare clinical manifestations or complex diagnostic challenges.

Exclusion criteria were: (1) studies focusing on male populations, (2) reports exclusively addressing complications (technical or post-intervention) of endovascular treatments (as these do not contribute to a primary gynecological clinical overview of PCS) and (3) articles without full-text availability.

### 2.3. Data Synthesis and Transparency

**Figure 1:**
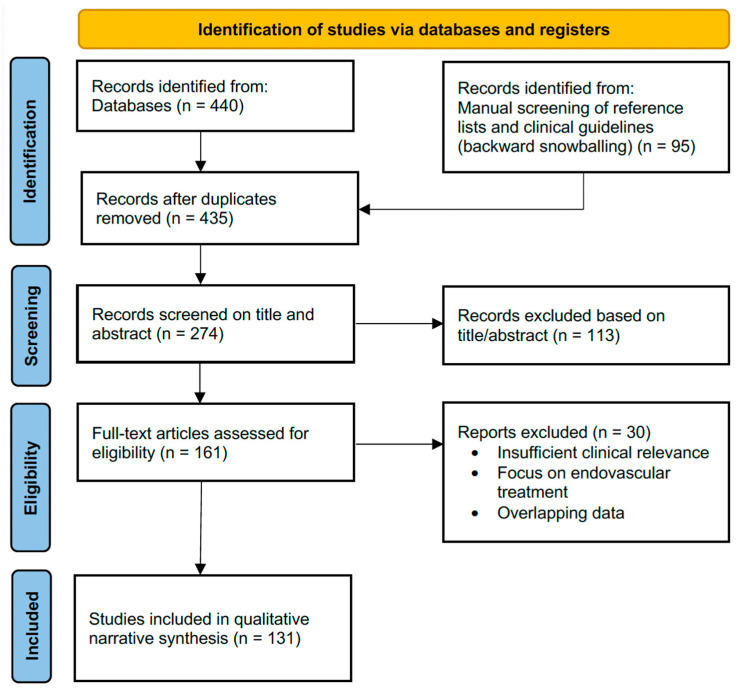
PRISMA-inspired flow diagram literature identification [17]. The identification phase integrated database searches (*n* = 440) and targeted backward snowballing from clinical guidelines (AWMF, ESHRE, UIP) and relevant reviews (*n* = 95). Following the removal of duplicates (*n* = 100), *n* = 435 records were screened. A total of *n* = 161 full-text articles were assessed for eligibility. Exclusion of reports (*n* = 30) was based on a lack of clinical gynecological focus or a primary focus on endovascular complications and interventional procedural details, resulting in the final inclusion of 131 sources.

A PRISMA-inspired flow diagram (Figure 1) illustrates the literature identification and selection process. After the removal of duplicates, records were screened by title and abstract, followed by a full-text assessment for eligibility.

### 2.4. Declaration of Generative AI

Following the journal’s guidelines, it is disclosed that generative AI was used solely for superficial text editing, including the improvement of grammar, spelling, punctuation, and the structural formatting of tables. The AI did not contribute to the analysis, discussion or clinical interpretation.

## 3. Anatomy and Pathophysiology

The exact cause of dilated pelvic veins in PCS is not fully understood. A combination of mechanical stress and hormonal factors leading to chronic venous insufficiency (CVI) and dilatation of the veins in the pelvis is suspected [18,19]. One of the main contributing factors appears to be congestion in the pelvic venous system [20].

### 3.1. Anatomy of the Pelvic Venous System

The pelvic venous network is highly intricate, variable, and well developed. In women, the primary veins responsible for draining the pelvic organs include the ovarian veins, as well as the common, external, and internal iliac veins. The internal iliac veins subdivide further into parietal and visceral branches [21]. Blood from the uterus drains via the uterine plexus into the left and right internal iliac, ovarian, internal iliac, and superior rectal veins [19]. Regular outflow from the pelvis, including the ovaries, drains via the ovarian venous plexus into the left and right ovarian veins [22]. The right ovarian vein empties directly into the inferior vena cava (IVC), while the left ovarian vein drains into the left renal vein before reaching the IVC [22]. This anatomical difference, particularly the acute angle between the left ovarian and renal veins, may contribute to the higher prevalence of PCS on the left side due to greater venous resistance on this side [22].

**Figure 2:**
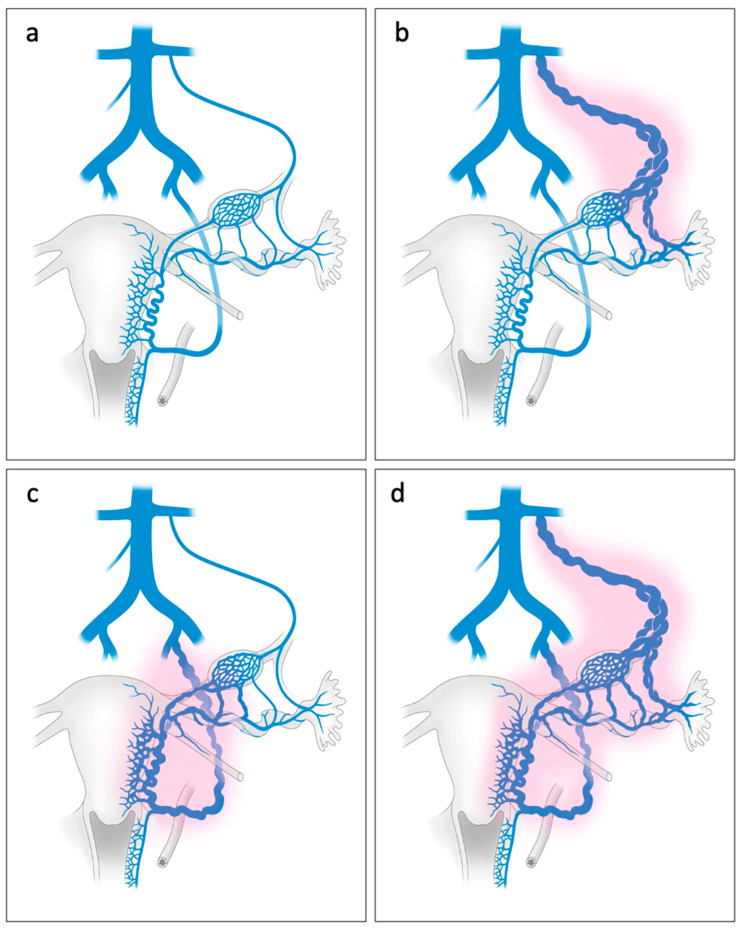
Anatomy of a normal venous system (**a**) and dilated pelvic veins due to PCS (**b**–**d**): PCS as seen most commonly on imaging studies of the ovarian veins (**b**), an isolated form of PCS with dilatation of the parauterine veins. (**c**) More complex but common form of PCS marked by dilatation of the ovarian and parauterine veins (**d**).

PCS and the resulting varicosities are not limited to the left ovarian vein [20]. Dilatation of the right ovarian vein occurs in 33% of patients with PCS [23]. Uterine venous dilatation is also common [20] (Figure 2c,d).

Although less frequently reported, varicosities surrounding pelvic nerves like the inferior hypogastric plexus and pudendal nerve may contribute to neuropathic pelvic pain, lower urinary tract dysfunction, and defecatory disorders [24]. This is supported by reports of pain relief after sacral plexus decompression, suggesting a role for neurovascular entrapment in PCS [25].

Special entities include the May–Thurner syndrome (chronic compression of the left iliac vein against the lumbar spine by the overlying right-sided common iliac artery [26]) and the nutcracker syndrome (left renal vein entrapment syndrome [26]), in which venous compression leads to venous dilatation distal to the site of obstruction and subsequent pelvic venous congestion [27,28]. Overall, however, PCS is primarily driven by venous reflux and dilatation caused by congenital or acquired valve insufficiency, with obstructive pathologies such as venous compression constituting less common contributing factors [12].

### 3.2. Histology

Postmortem anatomical studies have shown that the left ovarian vein lacks venous valves in about 13–15% of women, whereas the same is true of the right side in a mere 6% [29]. As in other regions affected by CVI, these veins exhibit increased expression of matrix metalloproteinases, which enzymatically degrade collagen and smooth muscle cells [30]. This may impair vasoconstriction, increase venous pressure, endothelial damage, and localized inflammation, ultimately resulting in chronic venous distension [19].

The histological features of pelvic varices resemble those of varicose veins elsewhere in the body, including fibrosis of the tunica intima and media, muscular hypertrophy, and proliferation of the capillary endothelium [31].

### 3.3. Risk Factors

#### 3.3.1. Hormonal Influence and Reproductive Age

Incompetent venous valves and enlarged venous diameters are more frequently observed in multiparous women and women of reproductive age [32]. PCS predominantly affects premenopausal women and its incidence declines significantly after menopause [13], unlike CVI of the lower extremities, which tends to increase with age [33]. This inverse pattern supports the hypothesis that estrogen and other hormonal influences contribute to venous dilatation and valvular incompetence in the pelvic vasculature.

#### 3.3.2. Estrogen and Ovarian Physiology

Women with PCS tend to have a larger uterus, a thicker endometrium, and polycystic changes in the ovaries [34]. These changes may resemble phenotypes seen in polycystic ovary syndrome (PCOS), with clusters of 4–6 cysts (5–15 mm in diameter) [35]. Animal studies have further supported this theory: female rats exhibit enhanced estrogen (E2/ER)-mediated venous relaxation [18].

The ovaries in women are exposed to estrogen and estradiol concentrations a hundred times higher than those in other venous territories [36], which may explain why the ovarian veins are primarily affected in PCS.

This leads to the theory that estrogen plays an important role in the genesis of PCS. It is unclear if the pelvic veins and ovaries are more sensitive to estrogen or have higher levels of estrogen [34].

The hormonal theory is further supported by the fact that PCS typically occurs in women aged 20–45 years, and that the symptoms can be alleviated by treatment with medroxyprogesterone acetate (MPA) [37].

#### 3.3.3. Multiparity

Multiparity is a well-established risk factor for PCS. Venous dilatation has been observed in over 63% of multiparous women, compared to only 10% of nulliparous women [36]. However, it should be noted that all patients in this particular study were asymptomatic [36]. During pregnancy, the capacity of the pelvic venous system can increase 60-fold compared to the non-pregnant state, significantly contributing to venous dilatation and valve incompetence [38]. In most women, venous diameter returns to normal postpartum, but this does not occur in patients with PCS [38,39].

This indicates, first, that venous dilatation alone is only one of several contributing factors in PCS, and second, that diagnosing PCS can be particularly challenging due to the lack of correlation between venous dilatation and symptoms [36].

While PCS is increasingly understood in vascular specialties, its recognition and diagnosis in Gynecology face challenges, including the absence of standardized non-interventional diagnostic tools.

## 4. Diagnosis of PCS

Despite CPP accounting for 10% of all gynecological consultations [2] and approximately 30% of these being attributable to PCS [7,8,9,11], little is known about its diagnosis in gynecological practice. Furthermore, the gold standard remains phlebography with contrast medium [40], which is an invasive procedure and is not available at the primary point of contact for PCS, namely, the gynecologist’s office.

### 4.1. Clinical Appearance

The diagnostic challenge of PCS lies in its wide range of symptoms. The time period from the onset of symptoms to diagnosis is still unknown. A similar time period, or even longer, may be assumed as that for endometriosis (approx. 10 years) [41].

Clinically, PCS usually becomes apparent during the evaluation of CPP. It is similar to the differential diagnosis of CPP, such as bladder dysfunction [42] and pain during sexual intercourse [43]. These symptoms are observed in many differential diagnoses such as adhesions and endometriosis (Table 1 and Table 2). However, there are also PCS-specific symptoms such as dull, dragging pelvic pain that worsens when standing or sitting; post-coital pain is pathognomonic for PCS [44].

The combination of tenderness on abdominal palpation on the ovarian region and a history of post-coital ache identifies PCS as a cause for CPP with a sensitivity of 94% and a specificity of 77% [12]. Multiparity, as mentioned, also appears to be a risk factor for PCS; morphologically dilated veins were identified via CT in 63% of multiparous women, compared to only 10% of nulliparous women [36].

### 4.2. Clinical Examination

During inspection the clinician should focus on varicose veins in general and the external genitalia in particular. Varicose veins occur more extensively in patients with PCS [45]. Similarly, signs of CVI in the rest of the lower extremity or hemorrhoids should be noted [46].

During bimanual palpation, a certain side-specific tenderness is observed. In women with PCS, approximately 91% showed side-specific adnexal tenderness on bimanual examination, compared to only 19% of women without PCS [7].

### 4.3. Ultrasound

TVUS is a non-invasive, cost-effective and widely available diagnostic tool in gynecological practice. When applied with appropriate expertise and knowledge, it is a valuable first-line diagnostic modality [40]. Ultrasound offers the significant advantage of demonstrating both endometriosis and PCS. Furthermore, it is able to indicate adhesions through the sliding sign and side-specific tenderness [47].

**Figure 3:**
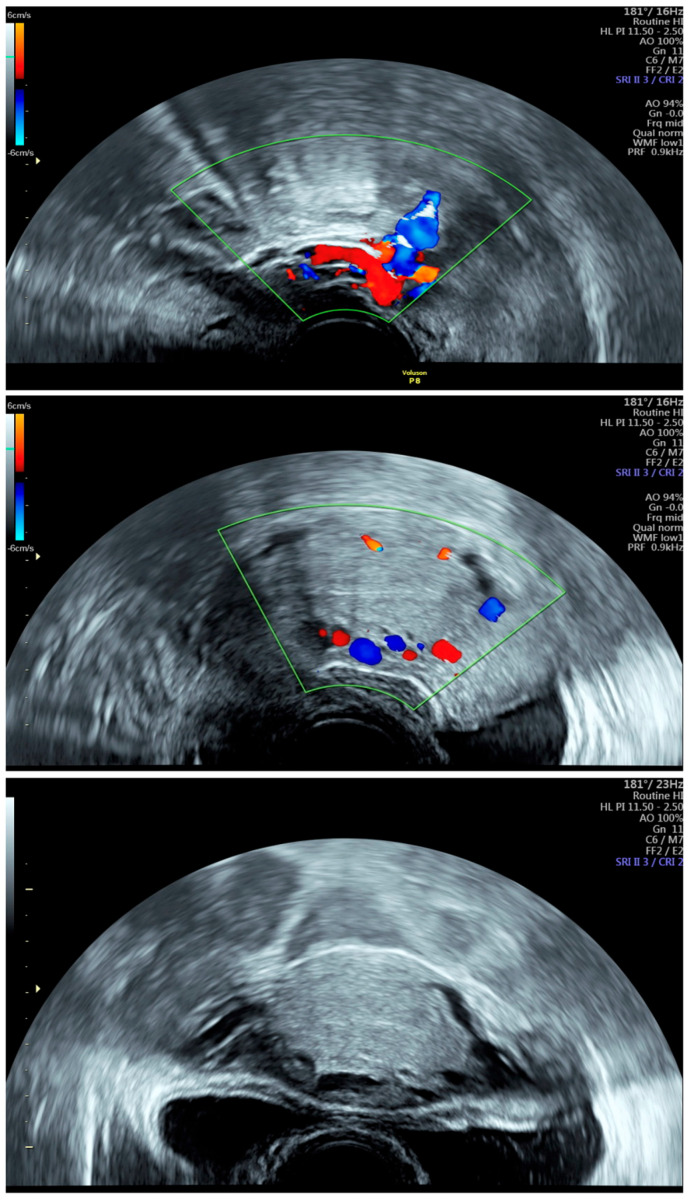
TVUS of a woman with PCS performed at our department, demonstrating dilated intramyometrial veins and prominent uterine veins with marked Doppler flow signals (UKSH Kiel, 2024).

Several ultrasonographic signs may serve as indicators of PCS [20,48] (Figure 3).

A vein dilatation of over 5 mm within the myometrium is indicative of PCS. Park et al. reported a positive predictive value for PCS outside the myometrium with a left ovarian vein dilatation of 6 mm of 83.3% [20]. In a meta-analysis, a cutoff at >7 mm in pelvic vein diameter (PVD) is suggested to provide better specificity, but a lower sensitivity than 6 mm [49].

Incidentally, polycystic ovaries also occur more frequently in the presence of PCS. Polycystic ovarian change was three times greater in patients with PCS than in the control group [35]. A further diagnostic tool is the detection of reflux using Doppler sonography. This is provoked by means of the Valsalva maneuver or, optionally, ultrasonography can be performed in a supine and anti-Trendelenburg position [50]. The sensitivity of this method is not as high as the measurement of vessel width, as reflux in the veins may also reveal different Doppler curves [51]. Two studies with small patient populations showed no significant difference in Doppler signals of individual veins compared to the control group [35,51]. Recently, Szodziak et al. demonstrated that transvaginal ultrasonography using a combination of four specific parameters achieves a sensitivity comparable to the gold standard of pelvic venography [40]. These parameters include dilatation of ovarian veins (cut-off diameter > 8 mm), low blood flow in ovarian veins (cut-off velocity <3 cm/s), reflux in ovarian veins (cut-off time > 1 s), and dilatation of arcuate veins within the myometrium. The combined assessment of all four parameters yielded a sensitivity of 100%, while the presence of even a single positive parameter still achieved a sensitivity of 94.5% [52].

### 4.4. Magnetic Resonance Imaging and Computed Tomography

Both Magnetic Resonance Imaging (MRI) and Computed Tomography (CT) are valuable imaging modalities for detecting PCS.

#### 4.4.1. Computed Tomography (CT)

CT is a useful, comprehensive and readily available tool for excluding other possible causes of CPP, especially malignant ones or vascular variants. On contrast-enhanced (CE)-CT, pelvic varicosities typically demonstrate attenuation characteristics similar to those of other abdominal veins on post-contrast imaging [53]. CE-CT typically shows dilated and/or varicose pelvic and ovarian veins. Most recent studies define ovarian vein dilatation as a diameter of 6 mm or more on axial images [36,54]. Due to the supine position during the examination, the extent of venous dilatation may be underestimated.

With due regard to radiation exposure [55], MRI should be given preference if available, particularly in premenopausal women [40]

#### 4.4.2. Magnetic Resonance Imaging/Magnetic Resonance Venography

Magnetic resonance imaging (MRI) offers excellent multiparametric visualization of the anatomical details of pelvic vessels and surrounding tissue [56], and rules out other possible causes such as deep infiltrating endometriosis (DIE), but is of limited accuracy for superficial endometriosis [57]. A simple enlargement of the pelvic veins is not sufficient for the diagnosis, as this is observed in a quarter of all women [58].

**Figure 4:**
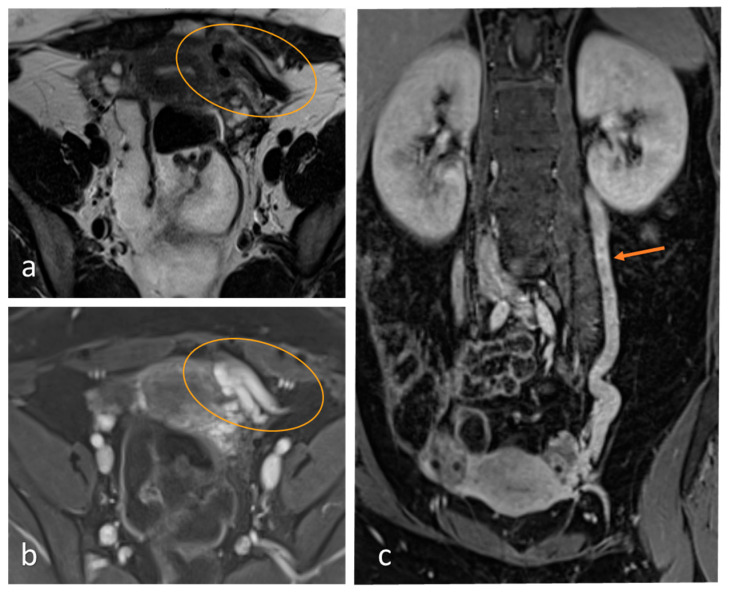
A 24-year-old woman presenting with left pelvic varicosis in the parauterine region (circle) and left ovarian varicosis, vessel diameter 10 mm (**c**): (**a**) transverse T2-TSE with prominent flow voids in the left parauterine aspect; (**b**) correlated transverse contrast-enhanced fat (UKSH Kiel, 2024).

**Figure 5:**
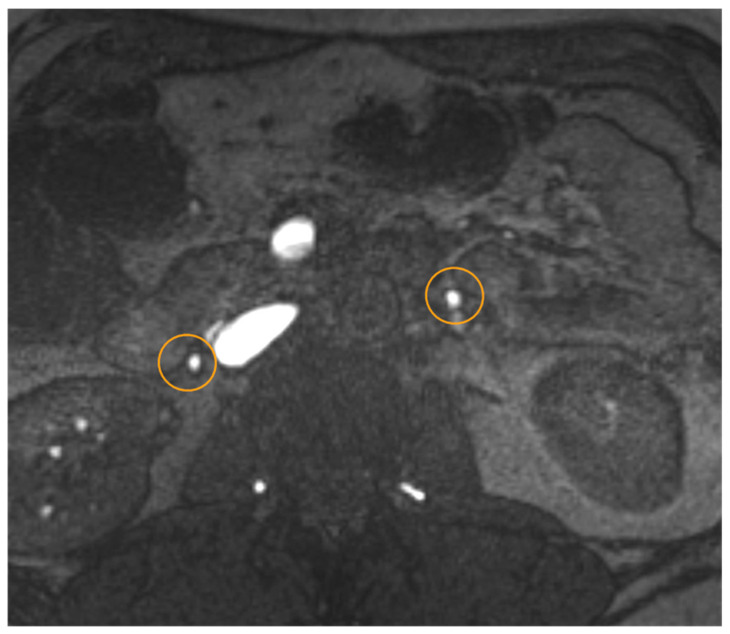
2D Time-of-flight (TOF) MR angiography: Transverse view in the mid-abdomen of a healthy patient. Note the lack of contrast fluid. Non-contrast TOF-MRA with a saturated band above the scan: While arterial flow is saturated, only venous flow in both ovarian veins (circle) is visible with the same signal intensities as in the IVC and superior mesenteric vein (no evidence of PVS; 3T Magnetom Vida, Siemens Healthineers, UKSH Kiel).

Contrast-enhanced magnetic resonance venography (MRV) permits time-resolved imaging of venous perfusion dynamics in the abdomen [59]. In comparison to catheter-based phlebography, MRI involves no radiation exposure. However, MRI is usually performed in a supine position, which may lead to under-diagnosis due to reduced venous congestion [56]. The dynamic visualization of veins, their dilated collaterals, and potentially hemodynamically relevant shunts to the internal pelvic vascular system are of great importance for defining a suitable interventional treatment approach [60]. Current technical innovations permit contrast-free MR angiography, which may improve sensitivity and image quality and involve shorter acquisition times. These procedures include time-of-flight MRA. (Figure 4 and Figure 5) [61,62].

The sensitivity and specificity of MRI techniques for detecting PCS with venography as a reference standard were 88–100% [56]. In another retrospective analysis, the sensitivity of time-resolved MR angiography for ovarian reflux ranged from 67% to 75%; the specificity was 100%; and the accuracy 79–84% [63].

### 4.5. Laparoscopy

Laparoscopy is commonly performed in women with unexplained CPP. Approximately 40% of all laparoscopies are performed due to CPP [4].

**Figure 6:**
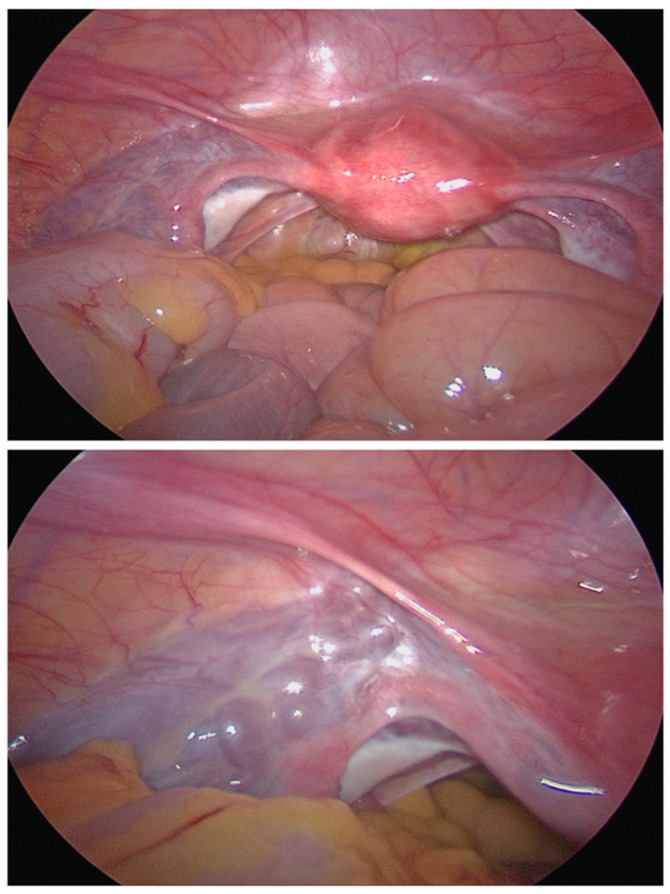
Intraoperative finding of PCS—A patient with CPP. The PCS appeared after restoring a neutral position during laparoscopy (UKSH Kiel, 2025).

Laparoscopic findings of prominent, enlarged, broad ligament veins may indicate pelvic varices and can be diagnostic for PCS (Figure 6) [64]. However, the supine position and compression of pelvic veins by intraperitoneal carbon dioxide may lead to false-negative findings [16]. Laparoscopic examination also allows for the exclusion of other potential causes of pelvic pain. Nevertheless, the sensitivity of laparoscopy for diagnosing PCS is only about 40% [64].

We lack data regarding intraoperative maneuvers that could enhance diagnostic accuracy. It remains to be established whether operating in a lower Trendelenburg position or reduced intraoperative CO_2_ pressure maintains surgical safety [65] and improves the visualization of pelvic varices [40].

### 4.6. Venography

Phlebography is the gold standard for the diagnosis of PCS [40]. A catheter is usually advanced transfemorally into the ovarian vein, common iliac vein, or internal iliac vein, and the respective venous plexus is visualized with contrast medium [7]. The ability to visualize reflux (Valsalva maneuver), collateral formation, and venous architecture in real time is unmatched by other diagnostic methods. The possibility of direct intervention is another major advantage of the method. Real-time visualization of functional collaterals is also unique to this diagnostic procedure. Thus, a holistic and functional overview of the individual collateral circuits can be obtained [66,67]. However, phlebography is an invasive diagnostic method. The risks include infection, bleeding, and contrast-induced nephropathy, all of which require careful consideration before use [40].

Major limitations of the procedure are its invasive nature and limited availability, which preclude its use as a first-line diagnostic tool. This highlights the need for a diagnostic modality that is both accessible and feasible at the initial point of care, specifically the gynecologist’s office [40].

### 4.7. Conclusion: Diagnosis of PCS

The diagnosis of PCS requires a multimodal approach that integrates clinical examination and imaging.

TVUS serves as the non-invasive, cost-effective first-line diagnostic tool, allowing for the simultaneous detection of endometriosis or adhesions. By combining specific ultrasound criteria, TVUS can achieve an accuracy nearing the gold standard [52].

Cross-sectional imaging (contrast-enhanced CT and MRI/MRV) offers essential complementary information for visualizing complex venous anatomy and collateral pathways, while excluding other causes of CPP. MRI is particularly preferred in premenopausal women due to its superior soft-tissue characterization and lack of ionizing radiation [40,55,58]. While laparoscopy remains valuable for excluding differential diagnosis of CPP, its sensitivity for PCS is limited by patient positioning and intra-abdominal CO_2_ pressure [4,5,16,64]. Transcatheter venography remains the diagnostic gold standard [40], providing definitive visualization of hemodynamics with the unique advantage of immediate intervention, though its invasiveness restricts it to second-line use [65,66,67].

### 4.8. Discussion: Diagnostics of PCS

Optimal diagnosis of PCS relies on a stepwise approach, starting in primary gynecological care to reduce diagnostic delays and distinguish PCS from other common causes of CPP such as endometriosis. A critical area for future clinical focus is the improved understanding of specific risk factors, including pregnancy-related thrombosis and postpartum venous insufficiency. Developing risk stratification tools based on these factors will be crucial for establishing preventive strategies. By addressing these diagnostic gaps, the management of PCS can become more consistent and efficient, ultimately leading to better long-term outcomes and an improved quality of life for patients.

## 5. Differential Diagnoses for PCS and CPP

The overlap with differential diagnoses for CPP, especially conditions like endometriosis [68], further complicates accurate and fast diagnosis.

### 5.1. The Problem with Endometriosis

In recent years, endometriosis has finally begun to receive the clinical and scientific attention it deserves in the field of benign gynecology. Endometriosis is a relatively common condition, affecting approximately 10% of all women of reproductive age [69]. However, not all women with endometriosis are symptomatic; a poor correlation was noted between the extent of disease and the severity of symptoms [70,71].

As a result, diagnostic steps for patients presenting with CPP may be primarily focused on endometriosis, which increases the risk of overlooking other potential causes, such as PCS. This diagnostic bias is further complicated by the frequent co-occurrence of both conditions [68]. One study reported that up to 80% of women with endometriosis also presented with dilated pelvic or ovarian veins, suggestive of PCS [68]. In other words, in a subset of women with endometriosis, symptoms of CPP may be partially or even primarily attributable to coexisting PCS. Moreover, studies have demonstrated pain relief in PCS following treatment with progesterone or GnRH analogs, similar to the therapeutic approaches used for endometriosis [11,72].

This overlap makes it particularly challenging to determine the precise etiology of CPP in clinical practice. Furthermore, there is limited evidence regarding a potential correlation between these two conditions, including whether one may predispose a patient to the other.

Despite their clinical relevance, PCS and endometriosis are frequently conflated or misdiagnosed [41]. This reflects a persistent gap in gynecological education and clinical awareness regarding the differentiation between these two conditions. To support clinical decision-making, we have created a comparative table summarizing the key symptoms and diagnostic findings observed during ultrasound and routine gynecological examination. It should be noted that we lack data about exact numbers in PCS (Table 1).

**Table 1:** How to distinguish between PCS and endometriosis.

	Endometriosis	PCS
Dysmenorrhea	90–100% [73,74]	Beginning 1 week before menses [74,75]
Dyschezia	39% of patients with DIE [73]	Rare
Dyspareunia	75%—deep, sharp, positional; cyclical [75]	71–78%—dull ache, non-cyclical, worse post-coitus (65%) [74]
Bladder	Dysuria in 19% of patients with DIE [73]	Hematuria [26], daytime frequency, incomplete voiding, and nocturia to 65% [42]
Sterility/Infertility	Strongly associated [76]	Multiparity [36]
Pain pattern	Cyclical, worse with menstruation [77]	Not mainly cyclical, also worsens after standing or intercourse [74,78]
Clinical bimanual exam	Uterosacral ligament nodularity and pain, retroverted or fixed uterus [77]	Pelvic tenderness, fullness; often nonspecific [12]
Clinical inspection	Usually normal; in deep disease: possible bluish nodules or tenderness in posterior fornix [77]	Usually normal, may reveal vulvar, vaginal, or cervical varices (bluish dilated veins) [74] hemorrhoids and signs of CVI [46]
Risk factors	Early menarche, short cycles, nulliparity, estrogen exposure [77], family history	Multiparity, prolonged standing, CVI, hemorrhoids [46,78]
Ultrasound findings	Endometriomas (ground-glass cysts), ovarian adhesions, adenomyosis, sliding sign, DIE [77,79]	Dilated/tortuous pelvic veins (>7 mm) [49], venous reflux in Doppler [20]

Abbreviations: PCS = Pelvic Congestion Syndrome; DIE = Deep Infiltrating Endometriosis; CVI = Chronic Venous Insufficiency; mm = millimeters.

### 5.2. Differential Diagnoses for CPP

In gynecological practice, awareness of the differential diagnoses of CPP is essential to avoid diagnostic delays, which may be as long as 33 years in cases of endometriosis [41]. In the following we summarize the most important gynecological (Table 2) and non-gynecological (Table 3) causes of CPP, and support clinical practice by providing a rapid orientation:

**Table 2:** Gynecological Differential Diagnoses for chronic pelvic pain (CPP).

Gynecological Differential Diagnoses for Chronic Pelvic Pain (CPP)					
Diagnosis	Incidence/Prevalence	Symptoms	Etiology	Treatment	Clinical Examination and Ultrasound
Endometriosis	Approx. 40,000/year in Germany: 33% of women undergoing laparoscopy for CPP [80]	Dysmenorrhea, dyspareunia, dyschezia and subfertility [81,82]	Endometrial tissue outside the uterine cavity and in myometrium. Many aspects still unclear. [80,83]	Medical amenorrhea (e.g., hormonal suppression) and/or surgery; multimodal pain management [80]	Vaginal sonography: sliding sign, adenomyosis, DIE nodes [79,84]
Adhesions/PID	18–35% following PID; 36% of women with CPP show adhesions on laparoscopic findings [85]	CPP; Prev. history of PID or surgery [86]	Adhesions with possible nerve ingrowth, unclear correlation between the severity of adhesions and pain [87,88]	Adhesiolysis–-effectiveness unclear. [85,89]	Previous history of PID or surgery [90], sliding sign [47,90]
PCS	Approximately 30% prevalence, relevant in multiparous women [7,8,9,36]	Dull pelvic pain and congestion, worsened after prolonged standing or intercourse [91,92]	Venous valve insufficiency, hormonal influences [37]	Venography, Embolization [40]	Signs of varicosis [93], dilated veins, reflux in TVUS [35]
Ovarian retention/remnant syndrome	84%with remaining ovarian tissue experienced pain [94,95]	Pain following hysterectomy or salpingo-oophorectomy [95]	Residual ovarian tissue after surgery causing pain [95,96]	Surgical removal of residual tissue [94,96]	No examination specifics or ultrasound findings
Fibroids	14.5% of women with CPP had fibroids [97]	Chronic lower abdominal pain, often accompanied by bleeding disorders [97]	Benign uterine tumors [97]	Medical treatment (e.g., GnRH analogs, hormonal therapy) or surgical removal (myomectomy, LASH) [98]	TVUS: hypoechoic, round/oval masses, uterine enlargement, posterior acoustic shadowing [98]
Vulvodynia/Vestibulodynia	Prevalence: 8–28% [99,100]	Burning, stabbing, itching of the vulva, pain on touch or penetration [101,102]	Somat. pain disorder in vestibulodynia involves neuromyogenic and psychosomatic components. trigger: *C. albicans* [101,103,104]	Multimodal therapy: psychotherapy, local treatment (topical anesthetics, corticosteroids), physiotherapy [102,105]	Cotton swab test [105]

Abbreviations: CPP = Chronic Pelvic Pain; PID = Pelvic Inflammatory Disease; PCS = Pelvic Congestion Syndrome; DIE = Deep Infiltrating Endometriosis; TVUS = Transvaginal Ultrasound; LASH = Laparoscopic Supracervical Hysterectomy.

**Table 3:** Non-Gynecological Differential Diagnoses for chronic pelvic pain (CPP).

Non-Gynecological Differential Diagnoses for Chronic Pelvic Pain (CPP)					
Diagnosis	Incidence/Prevalence	Symptoms	Etiology	Treatment	Clinical Examination and Ultrasound
Irritable Bowel Syndrome	Around 1% [106]	Chronic symptoms > 3 months (e.g., pain, bloating), gut-related [107]	Functional disorder with no identifiable organic cause [107]	Symptom-oriented therapy depending on symptoms (diet, stress management, medication) [107]	Diagnosis by exclusion [107]
Crohn’s disease/Ulcerative colitis	0.2% in Germany; 50% have CPP [108]	Lower abdominal pain, diarrhea, and weight loss [109,110]	Chronic inflammatory bowel disease (IBD), associated with HLA B27 [109]	Medical anti-inflammatory treatment; surgery if needed [109,110]	Ultrasound: wall thickening and signs of inflammatory activity in the colon are possible [110]
Celiac disease	1.4% worldwide [111,112]	Dyspepsia, constipation, flatulence, fatigue, depression [112]	Autoimmune reaction to gluten. With HLA-DQ2 [112]	Gluten-free diet [112]	Dermatitis herpetiformis, signs of vitamin deficiency [112]
Diverticulitis and SUDD	Older women; prevalence in women aged 70–85 years: about 50% [113]	Left-sided pain, acute abdomen in case of perforation [113]	Diverticular inflammation [113]	Change of diet, antibiotics, and surgery during the inflammation-free interval [113]	Hypoechogenic wall thickening over 5 mm with loss of wall layers [113]
Interstitial cystitis/BPS	52–500/100,000 women [114]	Pollakiuria, urinary urgency, and bladder pain [114]	Unclear, neurogenic and inflammatory components [114]	Increased fluid intake [114]	Diagnosis by exclusion [114]
Fibromyalgia	Common comorbidity in CPP [115,116]	Generalized pain, fatigue, and possibly RDS [116,117]	Functional, central sensitization [116,117]	Multimodal [116,117]	No specific findings on examination or ultrasound
Hernia/sciatic hernia	2% diagnosed by laparoscopy in women with CPP [118]	Local searing pain, pain on exertion [118]	Hernia sac protrusion due to tissue weakness [118]	Surgical repair [118]	-

Abbreviations: CPP = Chronic Pelvic Pain; Bowel Syndrome; IBD = Inflammatory Bowel Disease; SUDD = Symptomatic Uncomplicated Diverticular Disease; BPS = Bladder Pain Syndrome; RDS = Respiratory Distress Syndrome

## 6. Treatment of PCS

The treatment of PCS includes several approaches, such as analgesia, compression therapy, hormonal treatment, surgical intervention, and embolization.

### 6.1. Analgesia

Analgesic treatment should include non-steroidal anti-inflammatory drugs (NSAIDs) and pregabalin, which are recommended for generous use to manage symptoms until definitive interventions can be performed [40]. The addition of amitriptyline and gabapentin has been shown to significantly alleviate CPP [119,120].

### 6.2. Compression Therapy

The use of compression stockings for 14 days resulted in a significant reduction in symptoms in approximately 81% of women, as shown in a prospective study [121]. The administration of vasoconstrictors such as ergotamine also provided symptomatic relief in about 30% of cases [122].

Micronized purified flavonoid fraction (MPFF*) exerts a protective and tonic effect on the capillary and vein walls, and provides symptomatic relief in the mid-term [123]. In 20 patients, Simserk et al. (2007) observed a temporary improvement through the daily intake of MPFF 500 mg over six months [124].

### 6.3. Hormonal Treatment

Treatment with GnRH analogs was associated with a significant reduction in symptoms over a three-month treatment period [11]. However, it was associated with adverse effects, including perimenopausal symptoms such as hot flashes and mood disturbances. Furthermore, the duration of therapy is limited due to the increased risk of osteoporosis [11]. Reginald et al. investigated the use of MPA over six months in a population of 22 patients, demonstrating significant pain reductions of 75% in the median pain score. However, the symptoms recurred after discontinuation of the therapy [72]. Currently, we lack a clear recommendation for any of the aforementioned therapies [40].

Given the lack of robust clinical recommendations, conservative options like compression, hormones, and analgesia, should be viewed as temporary measures. Essentially, they function as a stopgap to manage symptoms while the patient awaits definitive surgical or embolic intervention [40].

### 6.4. Laparoscopy

**Figure 7:**
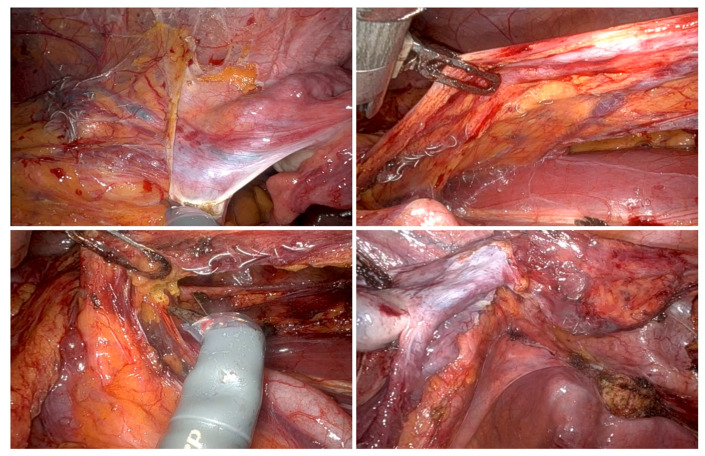
Intraoperative finding in a patient with recurrent PCS who was already receiving interventional coiling, undergoing complete resection of the left ovarian vein. (UKSH Kiel, 2024).

There is limited data on the use of laparoscopy for the surgical treatment of PCS. One study reported that patients who underwent bilateral laparoscopic transperitoneal ligation of the ovarian veins experienced pain relief; however, the follow-up period was limited to 12 months [125]. We do have case reports of ligation of ovarian veins leading to long-term symptomatic improvement [126,127], but found no data on recurrence rates or the need for reintervention (Figure 7).

Another study published in 1991 showed that bilateral oophorectomy combined with hysterectomy can be an effective treatment for CPP caused by venous congestion [128].

However, as the approach involves removal of the reproductive organs, it is regarded as a last resort. Laparoscopy is an invasive procedure that generally requires anesthesia and may be associated with significant morbidity, poor cosmetic outcomes, and a hospital stay of at least two days [129].

On the other hand, laparoscopy remains the most effective method for identifying other causes of CPP. Given its high co-incidence with endometriosis, the future of improving CPP treatment in a more holistic manner lies in the intraoperative diagnosis of CPP [68]. Randomized controlled trials (RCTs) are needed to investigate the simultaneous treatment of endometriosis and PCS in a single surgical session.

### 6.5. Endovascular Treatment

**Figure 8:**
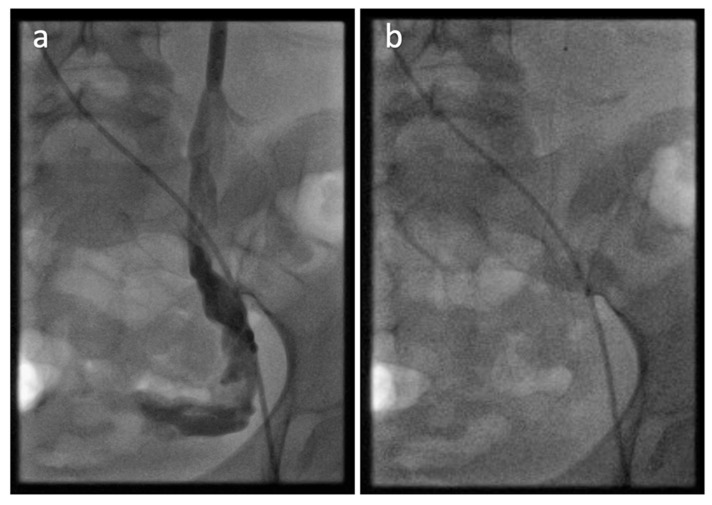
Pathological reflux in the left ovarian vein (**a**) with complete occlusion of the vessel after embolization (**b**) with 2 mL 2% Aethoxyskerol foam (UKSH Kiel, 2024).

Transcatheter embolization has emerged as one of the effective treatments for PCS. This minimally invasive procedure aims to occlude insufficient venous axes, re-route blood flow, and reduce pressure in the target veins [130]. Various embolic agents, such as metal coils, sclerosing agents, and gelatin sponges may be used [9]. Embolization is typically performed using a “sandwich” mixed technique which combines metallic devices with 2% Aethoxysklerol foam [9] (Figure 8).

The technical success rate of transcatheter embolization is high (99%) [91]. Significant symptomatic improvement has been reported in 88% of patients at 1–5 years of follow-up [91]. Studies using a visual analog scale (VAS) as a quantitative measure report statistically significant overall symptom improvement. On comparing post-treatment and pre-treatment values, the mean pelvic pain level had improved significantly from 7.6+/−1.8 before embolization to 2.9+/−2.8 after embolization [130].

While embolization is generally safe, complications may occur. The primary late post-procedural side effect is the recurrence rate of approximately 5% [40]. One important early complication is coil migration due to incorrect coil placement, which occurred in 1.4% of cases. Incidence rates differed from 2% to 4.2% [40]. Coil migrations are largely asymptomatic and usually require no additional treatment [40].

A further important complication is post-embolization syndrome, with occurs in 20% of cases. Symptoms include abdominal or lower back pain, subfebrile temperatures, nausea and bloating, which are usually self-limiting [40]. Reports of exacerbated symptoms after percutaneous treatment are rare [131]. Pain and post-embolization syndrome are more common when using sclerosing agents than when using coils [131].

Regarding the impact on hormone levels and ovarian function, current evidence suggests that pelvic and ovarian vein embolization does not result in significant alterations of basal female hormone levels, including follicle-stimulating hormone (FSH), luteinizing hormone (LH), or estradiol [130,131], and also does not significantly affect the menstrual cycle [58]. Furthermore, successful pregnancies have been reported after ovarian and pelvic vein embolization [130], indicating that the intervention does not markedly impair ovarian function.

However, the extent to which collateral venous pathways contribute to venous outflow after embolization remains unclear. Clinically, a five-year follow-up demonstrated low rates of symptom recurrence (8%) [60] and reintervention (3.9%), though 7–13% of patients experienced no clinical improvement [40]. The significant heterogeneity of the study design, however, limits our ability to draw definitive conclusions regarding long-term efficacy and outcomes [91,131].

A further limitation is that embolization alone may not sufficiently address multifactorial CPP. Given the high co-incidence of endometriosis, a laparoscopy may still be needed afterwards [68].

### 6.6. Conclusion: Treatment of PCS

The treatment of PCS encompasses a spectrum of conservative, surgical, and endovascular approaches [40]. While conservative and hormonal therapies may provide initial symptomatic relief, transcatheter embolization has emerged as the gold standard for definitive treatment, demonstrating high technical success rates, durable long-term symptom improvement, and a favorable safety profile without relevant impairment of ovarian function [40].

Data on the laparoscopic treatment of PCS remain limited, with evidence largely restricted to individual case reports and a single-center study reporting favorable outcomes [125,126]. Nevertheless, laparoscopy may play an important role when PCS is identified during diagnostic laparoscopy performed for multifactorial CPP [6].

### 6.7. Discussion: Treatment of PCS

Given the multifactorial nature of CPP, optimal management of PCS requires a multimodal and interdisciplinary approach.

Since PCS can be one of the underlying causes of CPP [5,14] and often coexists with conditions such as endometriosis, pelvic adhesions and other vascular abnormalities, multidisciplinary collaboration is particularly important [65]. Specialized CPP clinics equipped to address all major causes of CPP and maintain close links to interventional radiology could improve access to advanced diagnostic procedures such as pelvic venography and a wide range of therapeutic options, including surgery, hormonal treatment, analgesics, and embolization. A holistic patient-centered model involving gynecologists, interventional radiologists, pain specialists and mental health professionals, constituting a group of “pelvic experts”, would support individualized treatment and ultimately enhance the patients’ quality of life.

Future research should focus on randomized controlled trials to evaluate interventional, medical and combined treatment strategies, including the combination of embolization, hormonal therapy, surgical approaches and ovarian vein clipping. One possible approach would be an RCT enrolling patients with endometriosis and dilated pelvic veins with suspected PCS, comparing endometriosis resection alone versus endometriosis resection combined with ovarian vein clipping.

There is also a need to investigate hormonal and vascular markers such as AMH levels and estrogen receptor expression in ovarian veins, which may offer deeper insight into the pathophysiology of PCS. By addressing these therapeutic gaps, the management of PCS can become more consistent and efficient, ultimately leading to better long-term outcomes for patients.

## 7. Conclusions

PCS is a significant cause of CPP that requires significantly more attention in routine gynecological care. This review emphasizes that a multimodal diagnostic approach, led by transvaginal ultrasound and confirmed by venography, is essential for guiding therapy. While conservative options exist, transcatheter embolization remains the definitive treatment of choice. Ultimately, success in managing PCS depends on early identification in primary care and a multidisciplinary approach to distinguish it from co-existing pathologies like endometriosis.

## Data Availability

No new data were created or analyzed in this study. Data sharing is not applicable to this article.

## Abbreviations

The following abbreviations are used in this manuscript:

AI	Artificial Intelligence
AMH	Anti-Müllerian Hormone
BPS	Bladder Pain Syndrome
CE-CT	Contrast-Enhanced Computed Tomography
cm/s	Centimeters per second
CO_2_	Carbon Dioxide
CPP	Chronic Pelvic Pain
CT	Computed Tomography
CVI	Chronic Venous Insufficiency
DIE	Deep Infiltrating Endometriosis
E2/ER	Estrogen/Estrogen Receptor
FSH	Follicle-Stimulating Hormone
GnRH	Gonadotropin-Releasing Hormone
HLA	Human Leukocyte Antigen
IBD	Inflammatory Bowel Disease
IVC	Inferior Vena Cava
LASH	Laparoscopic Supracervical Hysterectomy
LH	Luteinizing Hormone
mm	Millimeters
MPA	Medroxyprogesterone Acetate
MPFF	Micronized Purified Flavonoid Fraction
MRI	Magnetic Resonance Imaging
MRV	Magnetic Resonance Venography
NSAIDs	Non-Steroidal Anti-Inflammatory Drugs
PCS	Pelvic Congestion Syndrome
PCOS	Polycystic Ovary Syndrome
PeVD	Pelvic Venous Disorders
PeVI/PVI	Pelvic Venous Insufficiency
PID	Pelvic Inflammatory Disease
PRISMA	Preferred Reporting Items for Systematic Reviews and Meta-Analyses
PVD	Pelvic Vein Diameter
RDS	Respiratory Distress Syndrome
s	Seconds
SUDD	Symptomatic Uncomplicated Diverticular Disease
TOF-MRA	Time-of-Flight MR Angiography
TVUS	Transvaginal Ultrasound
VAS	Visual Analog Scale
